# A Systematic Review of Multiple Family Factors Associated with Oppositional Defiant Disorder

**DOI:** 10.3390/ijerph191710866

**Published:** 2022-08-31

**Authors:** Xiuyun Lin, Ting He, Melissa Heath, Peilian Chi, Stephen Hinshaw

**Affiliations:** 1 School of Developmental Psychology, Faculty of Psychology, Beijing Normal University, Beijing 100875, China; 2 McKay School of Education, Brigham Young University, Provo, UT 84602, USA; 3 Department of Psychology, University of Macau, Macau 999078, China; 4 Department of Psychology, University of California, Berkeley, CA 94720, USA

**Keywords:** Oppositional Defiant Disorder, multiple risk factors, multi-level family factors theory, mediation, moderation

## Abstract

Oppositional Defiant Disorder (ODD) is characterized by a recurrent pattern of angry/irritable emotional lability, argumentative/defiant behavior, and vindictiveness. Previous studies indicated that ODD typically might originate within a maladaptive family environment, or was at least maintained within such an environment. As such, the present review summarized pertinent research from the last 20 years that focused on the pathways connecting family risk factors to the development of child ODD symptoms. A systematic search of electronic databases was completed in August 2020, resulting in the inclusion of 62 studies in the review. The review established a multi-level framework to describe the mechanisms underlying the pathway from familial factors to ODD psychopathological symptoms: (a) the system level that is affected by the family’s socioeconomic status and family dysfunction; (b) the dyadic level that is affected by conflict within the marital dyad and parent–child interactions; and (c) the individual level that is affected by parent and child factors. Additionally, from the perspective of family systems theory, we pay special attention to the interactions among and between the various levels of the pathway (moderation and mediation) that might be associated with the occurrence and severity of ODD symptoms. Considering future prevention and intervention efforts, this three-level model emphasizes the necessity of focusing on familial risk factors at multiple levels and the mechanisms underlying the proposed pathways.

## 1. Introduction

### 1.1. Oppositional Defiant Disorder

**Figure 1:**
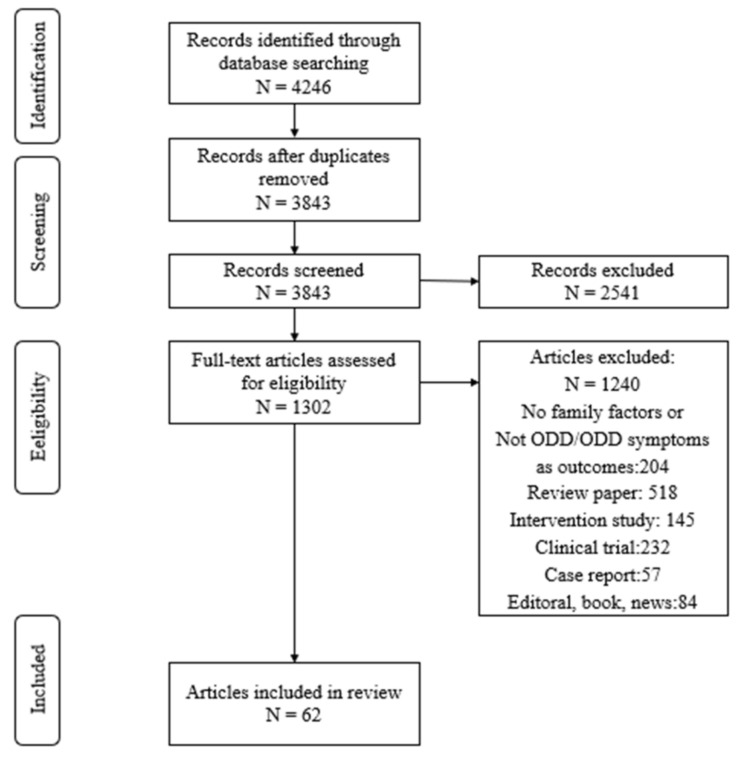
Flow diagram of study selection.

**Figure 2:**
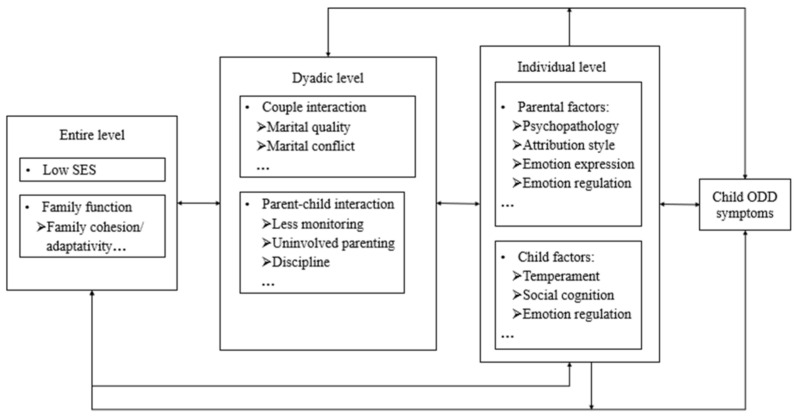
Three-level multiple family factors framework. Note that there also may be bidirectional relations and interactions among family factors and child ODD symptoms.

Oppositional Defiant Disorder (ODD) includes a variety of emotional and behavioral problems characterized by a recurrent pattern of angry/irritable moods, argumentative/defiant behavior, and vindictiveness toward authority figures [1,2,3] Although estimates of prevalence range from 1% to 11%, the average prevalence rate is believed to be approximately 3–4% [1].

Prevalence rates of ODD vary across populations. One study conducted in China found that the morbidity of ODD was 8% among Chinese children aged 7–15 years [4]. Furthermore, the rate of ODD may vary depending on the age and gender of the child [1]. For example, prior to adolescence, the disorder appears to be somewhat more prevalent in males than in females (1.4:1; [1]).

A growing body of research documents that ODD is associated with distress in the individual or others in his or her immediate social context (e.g., among family members, peer groups, work colleagues) and is accompanied by extensive social impairment [1,5]. Throughout their development, children with persistent ODD symptoms are likely to be involved in interpersonal conflicts [6,7], and also have a high risk for several adjustment problems, including antisocial behavior, impulsivity, substance abuse, anxiety, and depression [5,8]. Given the significant deleterious role of ODD in children’s social relationships [1,7], it is necessary to investigate factors that influence the emergence and trajectory of ODD. By deepening our understanding of this disorder, we can help to lay the groundwork to better inform prevention and intervention strategies. We propose that these strategies must strengthen family-based education, specifically focusing on the critical areas of marital conflict, parent–child relationships, and individualized child-focused supportive guidance [9].

### 1.2. ODD and Maladaptive Family Environment

Previous studies have identified numerous factors across diverse domains that appear to facilitate the development of ODD symptoms. These include demographic, biological, environmental, and individual factors and the interplay among these factors [5,8,10]. Due to strong biological correlates, numerous studies on ODD covered a wide variety of individual-level child factors that are associated with ODD symptoms. For instance, empirical studies have begun to explore its epigenetics and gene-environment interaction [11]. Neuroimaging findings converge to implicate various parts of the prefrontal cortex and amygdala [12]. Furthermore, alteration in cortisol levels has also been demonstrated consistently [13]. Additionally, psychosocial characteristics such as temperament, social cognition, and emotion regulation are strongly associated with child ODD symptoms as well [14,15].

Nevertheless, ODD has considerable environmental etiology [16]. Family factors, such as familial psychopathology, poor disciplinary practices, maltreatment, and neglect, are known to be significantly associated with children’s disruptive behaviors [1,14,16]. In fact, ODD is more prevalent in families where child care is disrupted by a succession of different caregivers or in families where harsh, inconsistent, or neglectful child-rearing practices are common [1,14,16]. Moreover, parenting practices, in particular, are the most amenable and easily approached target in the management of ODD, emphasizing the importance and necessity of investigating the links between family factors and child ODD symptoms. Therefore, the origin and development of ODD would not be completely understood if only individual child characteristics are considered. However, no systematic theory to our knowledge has been proposed to account for the occurrence and development of ODD within the family system, except for the multilevel family factors model [9].

### 1.3. Multilevel Family Factors

According to multilevel assessment, initially promoted by Vose (2010) and based on the family systems theory, the family is a dynamic and interactive system reflecting interdependent forces at multiple levels, including system, dyadic, and individual levels [17]. Further, Lin et al. (2018) proposed a multilevel family factors model to explain such effects (across the system, dyadic, and individual levels) on the development and maintenance/exacerbation of child ODD symptoms [9]. However, the family risk factors at each level require clarification and amplification.

With regard to the system level, the family is considered as a complete unit and system that is composed of surface characteristics (e.g., social economic status) and deep characteristics (e.g., family function). We review literature that describes both lower socioeconomic status and impaired family function as system-level family factors that are prominent contributors to child ODD symptoms. Of which, family function refers to the interaction of physical, emotional, and psychological activities among all family members [18]. As one of the system-level family factors, the realization of family function provides certain environmental conditions for the healthy development of family members in physiological and social aspects [18].

The dyadic level refers to the operation of each subsystem in the family, including the wife–husband subsystem and parent–child subsystem. Previous studies focusing on ODD risk factors were more inclined to explore dysfunctional parent–child interactions independent of other family-related interactions [19]. However, more recently, impaired couple interactions are shown to be a key contributor to the emergence and maintenance of child ODD symptoms [20]. As such, the current review identified literature that corresponded with the dual influence of couple interactions and parent–child interactions as family factors at the dyadic level.

For individual factors, we considered each family member as a separate subsystem. We included parental and child’s individual characteristics, cognitive factors, and emotional factors as factors at the individual level. According to this conceptual framework presented in this review, impairment and dysfunction of the family factors at three levels are critical for the occurrence and aggravation of child ODD symptoms.

Based on the multilevel family factors model [9], we review previous research, particularly exploring familial risk factors of ODD across the past two decades. Furthermore, we examine interactive mechanisms underlying these pathways, to better understand the development of ODD and to assist in developing effective family-based educational strategies for this disorder. We end the paper with a proposal of a three-level multilevel family factors framework to highlight the importance and necessity of understanding child ODD symptoms within the family context.

## 2. Method

Notably, the symptoms of ODD may be observed to some extent in individuals not formally diagnosed with this disorder. Longitudinal studies reveal that many ODD symptoms escalate from minor behaviors during the preschool period to more extreme behavioral patterns during adolescence [1]. Such longitudinal trends highlight the critical implication for early intervention that focuses on risk factors of both ODD symptoms and ODD-related behavior patterns [1]. All procedures and findings are reported in accordance with the Preferred Reporting Items for Systematic Reviews and Meta-Analyses (PRISMA) guidelines [21].

### 2.1. Literature Search

A systematic search of relevant electronic databases was completed in August 2020. We conducted a literature search in ProQuest, Google Scholar, and Web of Science using the search term ODD/oppositional defiant disorder/oppositional defiant disorder symptoms for all published journal articles from 2000 through 2020. Additionally, these articles’ content-related citations were also examined. This procedure yielded a total of 4246 articles (see Figure 1 for the flow diagram of study selection). Of these, we found that 1302 examined, to some extent, the associations of interest. Two independent coders independently screened these articles against seven inclusion criteria, as noted next.

### 2.2. Eligibility Criteria

Inclusion criteria were established a priori: (i) peer-reviewed articles; (ii) publication in English or Chinese; (iii) articles must be empirical studies; (iv) children or adolescents must meet diagnostic criteria for ODD or exhibit ODD symptoms (with any version of the DSM usable to assess the presence of ODD); (v) participant age under 18 years; (vi) consideration of ODD or ODD symptoms as a child outcome; (vii) inclusion of familial risk factors such as socioeconomic status and family function, marital conflict, parenting practices, parental psychopathology or emotion regulation, and child temperament or social cognition.

A total of 62 articles met all inclusion criteria and, thus, were eligible. Descriptive information about the studies is presented in Table 1.

## 3. Result

### 3.1. Family System Level

#### 3.1.1. Socioeconomic Status

Our review indicates the existence of increasing literature clarifying the negative effect of low socioeconomic status (SES) on child ODD symptoms [22,23,24,25]. For example, of a New Zealand longitudinal birth cohort, Boden, Fergusson, and Horwood (2010) sampled 926 youth who were diagnosed as CD (conduct disorder) or ODD. Family socioeconomic disadvantage was significantly associated with CD and ODD [26]. Similarly, in their longitudinal study—among a diverse community sample of 796 children—Lavigne and colleagues (2016) found that family socioeconomic status was significantly and negatively related to child ODD symptoms one and two years later (children at age 5 and 6, respectively) [19].

Life challenges associated with low SES include inadequate education, a chaotic family environment, and nonresponsive and/or harsh parenting. Altogether, the negative home environment prominently contributes to children’s externalizing and internalizing problems [27]. From this perspective, the mechanism by which SES affects ODD might be: a lower level of SES is linked to family conflict and hostility, which in turn contributes to more ODD symptoms in children [28]. Additionally, the family investment model proposed by Conger and Donnellan (2007) specifies that, when compared with children from low-SES families, children from higher SES families had more access to financial, social, and human (i.e., education) capital [29]. Accordingly, those parents might invest more in child-rearing activities to foster child academic and social success, a robust protective factor against child externalizing problems, including ODD symptoms [3].

#### 3.1.2. Family Function

A solid research base confirms the robust link between family function and child ODD symptoms [28,30]. Olson (2000) pointed out that family cohesion and adaptability are two core components of family function [31]. Family cohesion refers to the emotional connection among family members [32], while family adaptability refers to the ability of a family system to change its power structure, role relationships, and relationship rules in response to situational and developmental stress [31,33]. Lower levels of family cohesion and adaptability are prominently linked to an increased number of child ODD symptoms [28]. Compared to typical families, the quality of cohesion in families with ODD children was much lower [34]. In a more recent study, among a sample of 256 children with ODD (aged 6–12), Lin, and colleagues (2018) consistently found that a lower level of family cohesion and adaptability was correlated with more child ODD symptoms, while a higher level of family cohesion and adaptability predicted fewer child ODD symptoms [9].

According to McMaster’s family functional model theory, the failure of the realization of family basic functions in the process of operation might be conducive to various maladjustment and clinical problems among family members [35]. There are two possible explanations for this. First, poor family function might lead to family role confusion and unstable rules, which may contribute to the increase of physical diseases and mental disorders in children. Alternatively, families with poor function tended to have poor communication and coordination ability and were less likely to solve family crises, which would lead to the failure of the individual to learn positive coping styles. These threats to family functioning might cause or exacerbate the symptoms of ODD in children.

### 3.2. Family Dyadic Level

#### 3.2.1. Couple Interaction

There are considerable conceptual and empirical links between couple interaction (marital quality and marital conflict) and child ODD symptoms [23,36]. According to the emotional security hypothesis [37], marital conflict or a lower level of marital quality might create a negative emotional atmosphere in the family, and long-term exposure to such a negative emotional atmosphere may increase the risk of disruptive behaviors in children, such as ODD symptoms [38]. Additionally, based on the “spillover” hypothesis, the feeling or behavior born from one subsystem could emerge in another subsystem [39]. As such, parents with impaired marital functioning may engage in poor parent–child relationships, which in turn are related to poor child psychosocial outcomes [20].

In fact, previous studies have shown that lower levels of marital quality are associated with more ODD symptoms in children [20,40,41]. Conversely, adaptive marital relationships decrease the risk of child ODD symptoms [9,42]. Additionally, there is a consensus that marital conflict is a pivotal contributor to child ODD symptoms [14,23,26]. For instance, Burnette (2013) collected the data from wave 1 (*n* = 1992, mean age = 4.63 years) to wave 3 and the results suggested that intimate partner violence was associated with an increased risk of child ODD symptoms [43]. Similarly, in the 4-year longitudinal study (199 3-year-old children at wave 1), Harvey and her colleagues (2011) confirmed a significant predictive effect of intensive marital conflict on the number of child ODD symptoms [23].

#### 3.2.2. Parent–Child Interaction

As for factors associated with parent–child interaction, parenting practice appears to play a critical role in the development of child ODD symptoms. As such, in this study, we primarily focused on the effect of parenting practice on child ODD symptoms. Darling and Steinberg (1993) defined parenting practice as “the behaviors that include both the specific, goal-directed behaviors through which parents perform their parental duties and non-goal-directed parental behaviors, such as gestures, changes in tone of voice, or the spontaneous expression of emotion” (page 488) [44]. Maladaptive parenting practices that contribute to the development of ODD symptoms included less parental monitoring and less parental involvement and discipline [45,46,47,48]. Brown et al. (2017) investigated the association between parental monitoring and ODD in children at age 3 (*n* = 419) and again at age 6 [46]. The results manifested that poor parental monitoring at age 3 predicted more child ODD symptoms at age 6, suggesting that a higher level of parental monitoring is a potential protective factor for child ODD. An uninvolved parenting style was also significantly correlated with more ODD symptoms in children [45]. For example, by utilizing a community sample of 89 children ranging in age from 9 to 12 years, Pederson and Fite (2014) demonstrated that poor parental involvement is linked to more ODD symptoms [49].

Parental discipline practices have also been linked to child disruptive behavior disorder, including ODD. Specifically, inconsistent use of discipline, failure to use positive reinforcement (e.g., support and acceptance), and excessive use of corporal punishment have been linked to child ODD symptoms. Of which, inconsistent use of discipline refers to not following through with proposed punishments [50,51]. Tung and Lee (2013) sampled 162 5- to 10-year-old children and concluded that inconsistent discipline predicted elevated ODD among children experiencing low peer acceptance or high peer rejection, even controlling for children’s age, sex, number of ADHD symptoms, and parents’ race-ethnicity [51]. Additionally, less use of positive strategies, such as support and acceptance, in parenting also contributed to more ODD symptoms in children [43]. For instance, in a longitudinal study, Lavigne and colleagues (2016) found direct effects of parental hostility on child ODD symptoms one year later. In contrast, a higher level of parental support lowered the risk of subsequent ODD symptoms [19]. Moreover, research has shown that excessive use of corporal punishment promotes and exacerbates child problem behaviors and ODD symptoms as well [49,52]. Li, Lin, Hou, Fang, and Liu (259 6- to 13-year-olds; 2016) and Liu, Lin, Zhou, Zhou, Li, and Lin (368 7- to 14-year-olds; 2017) found that parental maltreatment served as a vital risk factor in relation to children’s emotional and behavioral problems [53,54]. Cruz-Alaniz, Martin, and Ballabriga (2018) also found that harsh parenting was positively associated with child ODD symptoms, based on data from their sample of 100 families with preschool children [55].

### 3.3. Family Individual Level

#### 3.3.1. Parental Individual Level of Factors

In this section, we review information about individual levels of parent factors. These factors include parental individual characteristics (parental psychopathology), cognitive factors (parental negative attribution style), and emotion factors (emotion regulation).

***Parental psychopathology***. Parent psychopathology, through its impact on the emotional climate of the family [37], is another factor linked to child ODD symptoms. To date, parental depression, aggression, anxiety, and alcohol and drug dependence have all been suggested to contribute to an increased number of ODD symptoms in children [56,57,58]. A study that investigated how parental depression affected children was conducted by Liu, Lin, Xu, Olson, Li, and Du (2017, [59]). Their research findings demonstrated that parental depressive symptoms were associated with higher levels of depression and conduct problems among children with ODD (*n* = 234, aged 6–13-years). Importantly, Harvey et al. (2011) found that initial changes in maternal depression corresponded to initial changes in child ODD symptoms [23].

Furthermore, Antúnez, Nuria, Granero, and Ezpeleta (2016) sampled a total of 550 children evaluated at ages 3, 4, and 5 and revealed that maternal high aggressive behavior is positively associated with the child’s ODD level [60]. Additionally, children of parents with depression and anxiety may be more likely to have behavioral problems and develop ODD. In a similar study, based on a community sample of 622 children who were assessed longitudinally at age 3 and age 5, Trepat, Granero, and Ezpeleta (2014) suggested that fathers’ anxiety-depression and aggressive behavior were strong predictors of child ODD [52].

In terms of parents’ alcohol and drug dependence, data from a community-based investigation of adolescents (age 17 years, *n* = 1252) and their parents revealed that parental alcohol and drug dependence were similarly associated with an increased risk for ODD [57]. Likewise, Rowe, Maughan, Pickles, Costello, and Angold (2010) also asserted that children with CD showed significantly higher rates of parental drug and alcohol problems when compared to their study’s no-diagnosis group [61]. The ODD group’s data fell between the other two groups (CD group and no-diagnosis group) when compared to the other two groups’ data. Rowe et al.’s analyses were based on four waves of data covering 1420 children in the community aged 9–16 years.

***Parental attribution style***. According to Weiner’s (1974) original attributional model, three proposed dimensions are linked to the attribution process [62]. These dimensions include the locus (whether the behavior was caused by the child, other people, or the environment), control, and stability [63]. Johnston and Ohan (2005) suggested that parents of children with ADHD and disruptive behavior disorders were more likely to attribute children’s negative behaviors as internal and stable, whereas they attributed children’s positive behaviors as external, less stable, and less controllable [64]. These attributions were especially noted when child behavioral stimuli were ambiguous. Parents’ inaccurate attribution of their child’s ODD symptoms frequently limited the parent’s ability to realistically interpret the child’s real intent underlying the behaviors. In turn, the parent’s negative attributions further impacted child development negatively [64].

Research on the relationship between parental attribution style and child ODD symptoms is less abundant. However, studies on the linkages between parental attributional style and children’s emotional and behavioral problems may provide indirect evidence. For example, Wang and Wang (2018) sampled 864 students (mean age = 13.55 years) in China and found that negative paternal attribution was positively associated with child emotional problems [65]. From the perspective of parental locus of control (PLOC), McCabe, Goebring, Yeh, and Lau (2008) studied 58 children with behavior disorders and 57 typically developing children with no behavioral disorders. Children in the sample ranged between the ages of 3 and 7 [66]. They found that their sample of Latino parents were more apt to adopt external control attribution and that their pre-school children were more likely to exhibit behavioral problems. In studies such as McCabe et al. (2008) and Wang and Wang (2018), parents of children with ODD symptoms are inclined to consider child behavior problems as uncontrollable and difficult to manage. Consequently, these parents might not take the initiative to discipline children.

***Parental emotion factors***. One of the main contributing factors to the development of ODD symptoms in children is parental socialization of emotion, which is thought to occur through modeling of emotional expression and regulation, direct coaching in how to identify and cope with emotion, and/or parental reinforcement of emotional expression [67]. Dunsmore, Booker, and Ollendick (2013) illustrated that parental emotional expression placing value on children’s appropriate expression of emotion, and engaging in direct instruction about coping strategies, may ameliorate children’s emotion regulation and emotional understanding, which would reduce ODD symptoms [68]. In the aspect of the empirical research, Duncombe, Havighurst, Holland, and Frankling(2012) conducted a study among 373 5- to 9-year-old children with typical ODD symptoms. They found that negative parental emotional expression positively correlated to the number of ODD symptoms in children [56]. Similarly, by using a longitudinal design with a sample of 146 children (5 years old at Time 1) and their parents, Weber-Milne (2015) confirmed the strong link between parents’ emotional expression and children’s OD behavior [69].

As for the other aspect of parent emotion socialization, parental emotion regulation plays an important role in the development and maintenance of child ODD symptoms as well [53]. The deficits in parental emotion regulation might affect the process of child emotion socialization, contributing to poor psychological outcomes [70]. For instance, among a sample of 239 6- to 13- year-old children with ODD, Lin et al. (2019) found that parental emotion dysregulation was positively associated with child depressive symptoms [15]. Additionally, Jiang, Lin, Zhou, Hou, Ding, and Zhou (2020) sampled 123 Chinese children with ODD (ages 6–13) and their mothers. Their data indicated that maternal emotion dysregulation was significantly and positively related to child ODD symptoms [71].

#### 3.3.2. Child Individual Level of Factors

Over the past 20 years, research findings indicate that several individual child factors are associated with the development of child ODD symptoms. These child factors include child individual characteristics (children’s temperament), cognitive factors (social cognition), and emotion-related factors (emotion regulation). These factors are described in the following sections.

***Children’stemperament.*** Researchers have focused on the link between infant temperament—such as high novelty seeking, low harm avoidance, high persistence, negative affect, low levels of effort control, and disinhibition [72,73]—and future psychopathology, including ODD [74,75].

Kim, Cho, Kim, Kim, Shin, and Yeo (2010), for example, confirmed the positive relationship between novelty seeking and child ODD symptoms. Their study was conducted with parents of children (mean age of children, 10.4 ± 3.0 years), including 94 parents of children with ODD and 94 parents of children with no identified behavioral problems [76]. Kim et al.’s study is in line with other research, such as the study conducted by Joyce and Oakland (2005) [77]. Interestingly, Melegari and her colleagues (2015) elucidated that children with ODD were characterized with higher scores on novelty seeking, persistence, and harm avoidance. In particular, higher persistence accounted for more resistance to the extinction of maladaptive behaviors, which align with the oppositional and defiant symptoms in ODD. Their sample consisted of four groups (*n* = 120; 30 per group): ADHD, anxious, ODD, and control children. The mean age of children was 4.65 ± 0.88 years. [78]

Additionally, a high level of negative affect (i.e., high levels of anger, sadness, and fear) proved to be a robust risk factor for child behavior problems, especially when combined with a lower level of effortful control (i.e., thoughtful, deliberate forms of regulation; [60,69]). Nielsen (2014) found that negative affect was positively correlated with the initial level of ODD symptoms, and predicted an increase in ODD symptoms from age 4 to 6 (*n* = 797) [79]. While high effortful control was associated with minimal ODD symptoms at age 4, effortful control did not predict a change in such symptoms over time. However, in interactions with negative affect, the protective effect of effortful control was strong for children high on negative affect but lower for children low to moderate on negative affect. More recently, Frick and Brocki (2019) sampled 77 children aged 8 to 12 years and found that child effortful control contributed independently to inattention and hyperactivity/impulsivity, while negative affect contributed to child ODD symptoms [80].

Furthermore, disinhibition (i.e., difficulties with behavioral, cognitive, and emotional regulation), another similar dimension of maladaptive temperament, has also attracted researchers’ attention [76]. Extant studies have indicated that disinhibition is a prominent contributor to the development of children’s disruptive behavior disorders, including behaviors associated with ODD [81]. Additionally, among a total of 7140 children in a longitudinal study, Stringaris et al., (2010) found that high levels of emotionality and activity at the age of 38 months strongly predicted the development of ODD at the age of 91 months [75]. In this sense, higher levels of activity might contribute over time to children developing additional ODD symptoms, including impulsivity and hyperactivity. Notably, over time, higher levels of emotionality might lead children to behave in an oppositional manner, particularly during stressful or emotionally arousing situations [82].

***Children’s social cognition.*** Previous studies have investigated the relationship between social cognition and children’s emotional and behavior problems, including ODD symptoms [83,84,85,86]. For instance, research conducted by Dinolfo and Malti (2013) has verified that interpretive understanding, sympathy, and strength of moral emotion attribution predicted ODD symptoms negatively [87]. Their study included an ethnically diverse sample of 128 4- and 8-year-old children. Additionally, Osa, Granero, Domenech, Shamay-Tsoory and Ezpeletain (2016) sampled 538 preschoolers, more specifically in a subsample of 40 children diagnosed with ODD [88]. The results revealed that children diagnosed with ODD had a slower response time when performing the affective mentalizing condition than children without the disorder, indicating that children with ODD were impaired in the theory of mind aspect and the psychological domain. Furthermore, Skoulos and Tryon (2007) studied 27 children who met the ODD diagnostic criteria and 27 children (aged between 14.3–19.3 years) who did not meet the ODD diagnostic criteria [89]. They suggested that a lack of adaptive social skills exacerbated psychopathology in adolescent females who were identified with educational disabilities and who displayed symptoms of ODD.

Additionally, the social information processing (SIP) was particularly well documented in previous studies, which accounted for the proximal factors of child externalizing problems [90]. Specifically, there are several steps in the SIP model (encoding, making attributions, selecting a goal, generating responses, evaluating responses, and enacting responses), and researchers investigated the different outcomes resulting from problems associated with different SIP steps and the interactions among these steps [90]. Coy, Speltz, Deklyen, and Jones (2001) compared the difference in social information encoding between children with ODD and those without ODD [91]. The sample included 88 preschool boys with ODD and 80 nondisruptive boys, with longitudinal assessments over a two-year period. The results showed that boys with ODD encoding of social information were less accurate than normally developing children.

***Children’s emotionregulation.*** The development of adaptive emotion regulation (ER) competencies is critical in children’s early development [92]. When persistently failing to cope with negative emotions (i.e., venting and lack of effective regulatory strategies), children become overwhelmed with distress and frustration and are at a heightened risk for developing psychopathology and experiencing maladaptive outcomes [93,94]. Indeed, considerable research has shown that maladaptive ER competencies are significantly related to the development of child ODD symptoms [95,96,97]. More succinctly stated, children with impaired ER competencies appear to be more vulnerable to developing ODD symptoms and other psychopathology [98,99,100,101].

In another study conducted by Schoorl, van Rijn, de Wied, van Goozen, and Swaab (2016), included 65 boys with ODD/CD and 38 typical developing boys (8–12 years), Schoorl et al. (2016) asserted that the ODD/CD group rejected more ambiguous offers than the non-clinical (NC) group, which was seen as an indication of poor emotion regulation [102]. Parents also reported that the ODD/CD group experienced more emotion regulation problems in daily life than the NC group. Additionally, Paliziyan, Honarman, and Arshadi (2018) sampled 320 students with a mean age of 16.34 (*SD* = 0.66) years and found that emotion dysregulation was the most effective predicting variable of ODD [103]. More recently, Lin et al. (2018, 2019) also stated that lower levels of emotion regulation play a transdiagnostic predictive role in children’s co-occurring internalizing psychopathology and ODD symptoms [9,15].

### 3.4. Multi-Level Family Factors Interactions

Taken together, the multilevel family factors, including the system level, dyadic level, and individual level, play critical roles in the development of child ODD symptoms. With regard to the system level, both low levels of SES and family dysfunction were related to the development of child ODD symptoms. As for the dyadic level, the negative effects of marital conflict and negative parent–child interactions on child ODD symptoms is well documented. Concerning the individual level, both parental and child factors (individual characteristics, cognitive factors, and emotion factors) contribute to the development and maintenance of child ODD symptoms.

Nevertheless, according to family systems theory [104], during interactions, any changes in the function of one family member elicits a compensatory change in another family member. Within the family system, both the interactive processes among different familial factors are emphasized. In other words, rather than in a direct manner, the influence of risk factors in one family level on the development of child ODD are likely to be mediated and moderated by factors in other levels of the family system.

Given the importance of the family systems theory, in the following sections, we review the interplay of how family factors at the system, dyadic, and individual level affect child ODD symptoms. We elucidate these interconnected multi-level relationships as we propose our understanding of how factors at system, dyadic, and individual levels exert effects on the development and maintenance of child ODD symptoms.

#### 3.4.1. Mediation/Moderation Effect between Family SES and Child ODD Symptoms

Researchers have illuminated that lower SES, as a distal factor to the child, exerts its effect indirectly, through more proximal factors, such as dyadic marital relationship, parent–child relationship, and parenting, as well as individual parent and child factors [19,28]. For example, Granero, Louwaars, and Ezpeleta (2015) sampled 622 3-year-old children and demonstrated that the association between low SES and high ODD was partially mediated by difficulties in child effort control, corporal punishment, and inconsistent discipline [105].

Additionally, Lavigne et al. (2012) studied 796 4-year-old children and found a direct and negative relationship between SES and child ODD symptoms [28]. Further, this relationship was mediated by dyadic-level factors (e.g., marital conflict, parental hostility, parental support, and parental scaffolding), and individual-level factors (e.g., child effortful control and sensory regulation). Consistently, Lavigne et al. (2016) examined a cascade model of ages 4 and 5 multi-domain factors on child ODD symptoms at age 6 in a diverse community sample of 796 children [19]. Significant indirect effects on age 6 ODD symptoms were found for age 4 SES via age 5 conflict and parental scaffolding skills.

#### 3.4.2. Mediation/Moderation Effect between Family Function and Child ODD Symptoms

Aside from SES, family dysfunction, a system-level family factor, also appears to predict child ODD symptoms through the mediation of dyadic- and individual-level factors, such as parenting practices and parental psychopathology [28]. In fact, impaired family function might lead to more negative parenting practices (e.g., inconsistent discipline and hostile parenting) and fewer positive parenting practices (e.g., warm and supportive parenting), which further facilitates child ODD symptoms [28].

Individual parent and child factors are also significant mediators between family dysfunction and child ODD symptoms. For example, studies have suggested that family stress and conflict, associated with parental depressive symptoms and child temperament, may facilitate child ODD symptoms [28]. Additionally, Lin et al. (2018) purported that family cohesion/adaptability affected child ODD symptoms indirectly through the sequence of parent–child relationship and child emotion regulation [9]. This study’s results illustrated that the distal factor of family cohesion/adaptability could exert its effects on child ODD symptoms via more proximal factors, such as parent–child relationship and the child’s emotion regulation.

Moreover, individual child factors also moderated the pathway from family dysfunction to the development of ODD symptoms. For instance, Chen et al. (2020) demonstrated that children’s emotional lability/negativity significantly moderated the link between family violence and children’s ODD symptoms, by using a sample of 409 children (*M*age = 9.36, *SD* = 1.55). Consistent with this viewpoint, a higher level of family violence was associated with higher levels of ODD symptoms among children with lability/negativity [98].

#### 3.4.3. Mediation/Moderation Effect between Couple Interaction and Child ODD Symptoms

Several studies have explored the individual mediators and moderators between marital conflict and child ODD symptoms [15]. In particular, Lavigne et al. (2012) conducted a multi-domain model of risk factors for ODD symptoms in a community sample of 796 4-year-old children [28]. The results showed that marital conflict had both direct effects on ODD symptoms, and indirect effects via parental depression and child effortful control and sensory regulation.

Furthermore, Ding et al. (2019) found that the link between marital quality and child ODD symptoms was particularly moderated by child gender [20]. Specifically, parental marital quality predicted subsequent ODD symptoms for boys. However, the direct effect of paternal marital quality on girls’ ODD symptoms was not significant. Their sample included 253 6- to 13-year-old children with ODD and their parents and teachers from mainland China.

#### 3.4.4. Mediation/Moderation Effect between Parent–Child Interaction and Child ODD Symptoms

Previous studies also explored the interactive effect between parent–child interaction and other familial risk factors on the etiology of child ODD symptoms [28,69,71]. Numerous individual parent and child factors were identified to mediate or moderate the link between parent–child interaction and child ODD symptoms [19,68]. For example, Duncombe et al. (2012) elucidated that children’s emotion regulation ability may mediate the spillover from parenting practices to child ODD symptoms [56]. In line with this, Lin et al. (2018) found that parent–child relationships contributed to the development of child ODD symptoms through the mediation of child emotion regulation [9]. More recently, Lin et al. (2019) found that harsh parenting practices were directly and indirectly related to child ODD symptoms through child emotion regulation [15]. Additionally, parental emotional abuse of the child was associated with child depressive symptoms directly and indirectly through child emotion regulation. In a longitudinal study, Jiang et al. (2020) asserted that children’s wave 3 emotion dysregulation mediated the longitudinal associations between mother–child relationship quality and children’s wave 3 ODD symptoms [71].

Furthermore, children’s emotion regulation has also been verified as a moderator to the parent–child interaction and ODD symptoms [68]. Dunsmore and colleagues (2013) used a sample of 79 parent–child dyads (children’s age ranged from 7 to 14 years) and illuminated that when children were high in emotion lability/negativity, mothers’ emotion coaching was associated with fewer child ODD symptoms, supporting the potential of maternal emotion coaching as a protective factor for children with ODD, especially for those high in emotion lability [68]. Additionally, children’s specific temperament might moderate the link between parent–child interaction and child ODD symptoms. For instance, prior research has indicated that the more a father responded sensitively to the child, the less likely the child was to display OD behavior and this relationship was stronger the angrier a child’s temperament [69]. Moreover, Burnette (2013) clarified differences between factors affecting ODD symptoms in girls and boys [43]. Specifically, the significant link between parental physical abuse and ODD symptoms was found in girls but not boys. However, parental emotional responsiveness was a significant predictor for boys only.

#### 3.4.5. Three-Level Multiple Family Factors Framework

Drawing upon the existing research, we developed a three-level multiple family factors framework that integrates the multilevel family factors model [9] and aforementioned family risk factors, and their interactions (see Figure 2). It is important to note that causality is likely bidirectional: Children’s ODD symptoms influence family factors at different levels as well as vice versa [71,106]. Due to the space limitation and our primary focus of the model (i.e., the hierarchy of family factors at different levels and their effects on child ODD symptoms), the mutual linkages are not further elaborated in this review.

The three-level multiple family factors framework provides groundwork for research to better describe the interplay of family risk factors at system, dyadic, and individual levels and explain how these interactions affect the emergence and exacerbation of child ODD symptoms in the family system. Additionally, the three-level multiple family factors framework highlights the importance and necessity of understanding child ODD symptoms within the family context.

#### 3.4.6. Future Prevention and Intervention Efforts

This multilevel family systems theory prominently expands our understanding of the occurrence, maintenance, and development of child ODD symptoms. Based on our broadened perspective, rather than viewing ODD symptoms as solely and independently arising from children’s individual dysfunction, we propose that child ODD symptoms are embedded in and influenced by multiple levels of family factors (the system, dyadic, and individual level). Because child ODD symptoms undoubtedly hinge on comprehensive family matters, family members must consider their role in and responsibility for child ODD symptoms. This proposed multilevel family factors theory opens a more systematic and global view to explore prevention and intervention strategies.

An accumulation of evidence suggests that intervening with ODD is a comprehensive family matter [107]. Additionally, intervention strategies with multiple components are inclined to more effectively and promptly eliminate or decrease child ODD symptoms [108,109]. As such, future intervention and prevention efforts should carefully consider how family factors at multiple levels are related to child ODD symptoms, then target multiple levels in the family system, rather than solely focusing on child dysfunctional characteristics.

## 4. Discussion

Based on publications in the past two decades, we summarized familial risk factors which are implicated in the etiology and maintenance of child ODD symptoms. Based on this summary, we developed a multiple-level framework describing the mechanisms underlying the pathway from familial risk factors to ODD psychopathological symptoms. Particularly, from the perspective of the family systems theory, we divided these familial risk factors into three different levels, including the system level, dyadic level, and individual level (see Figure 2). From this perspective, children are at the highest risk for developing ODD symptoms when confronting prominent problems in system level (e.g., lower SES, family dysfunction), dyadic level (e.g., marital conflict, poor parent–child interaction), and individual level (i.e., individual parent and child characteristics, cognitive factors, and emotion factors).

We also explained the underlying mechanism between familial risk factors and child ODD symptoms. Simply stated, there are significant interactive effects among various familial risk factors, which serve as both mediators and moderators in this process. Specifically, indicators of the system level are the most distal risk factors of child ODD symptoms in the family system, and the link between system level factors and child ODD symptoms are prone to be mediated and moderated by factors at dyadic and individual levels. Indicators on the dyadic level are more likely to be associated with child ODD symptoms directly and indirectly via individual-level factors. Moreover, indicators on the parent and child individual level are the most proximal risk factors of ODD symptoms and are inclined to contribute to child ODD symptoms in a direct manner.

Overall, our study outlined the direct and indirect influences on the development, maintenance, and modification of child ODD symptoms, including overall family functioning, interparental relationship, parent–child relationship, parent characteristics, and child characteristics. Based on this information, we recommend that future research focus on multiple levels of familial risk factors related to child ODD symptoms, rather than focusing on individual child psychological functioning. Additionally, our review provides contextual information to guide the development of more effective prevention and intervention strategies.

Throughout the review, we found multiple levels of risk factors in the family system that contributed to the occurrence and development of child ODD symptoms [5]. These multiple levels of risk factors included factors of impaired family socioeconomic status (SES) and family dysfunction on the system level; factors of marital conflict, maladaptive parent–child relationships, and poor parenting practices on the family dyadic level; and factors of parent and child characteristics on the individual level. In addition to these factors documented in current review, we also believe that there are still more factors linked to ODD symptoms. Logically, this suggests that ODD is a multiple-risk-factor consequence rather than a single-child-individual problem.

Taking both parent–child interaction and child individual factors into consideration, the three-level multiple family factors extends Vose’s (2010) work, which proposed a multiple-level assessment of family functioning [17], involving whole family, dyad (i.e., interparental relationship), and parental individual levels. Specifically, in the three-level multiple family factors framework, we expanded on the family factors at the dyadic level. In addition to interparental relationship, we also include the parent–child relationship. Additionally, for family factors at the individual level, in addition to parental individual factors (including both paternal and maternal individual factors), we include child individual factors.

In this review, our proposed three-level multiple family factors framework urges researchers, practitioners, teachers, and parents to conduct prevention and interventions for child ODD symptoms from the perspective of multilevel family factors theory. That is, we must focus on risk factors of different levels, not solely on child individual factors. Furthermore, for children with ODD symptoms, addressing multiple factors in the family system will prove more effective than treating children in isolation of their environment, most importantly, the family context. Our proposed framework also clarifies the interactive effects among various familial risk factors, by examining the influences of both mediators and moderators. Particularly, research that investigated factors at the system level was limited. The majority of studies highlighted the mediated role of the dyadic and individual levels of factors in the pathway from system-level risk factors to child ODD symptoms. Furthermore, factors at the dyadic level were extensively examined. Our review addresses the important roles of marital conflict and impaired parent–child interaction at the dyadic level in the mechanism underlying the etiology of child ODD symptoms [23,45].

Additionally, it is important to note that, factors at the dyadic level may function in two different ways in the family process [28]. First, factors at the dyadic level are related to child ODD symptoms directly and indirectly via individual parent and child factors. Second, dyadic-level factors serve as important mediators linking the system level factors and child ODD symptoms. Moreover, according to our review, parental and child factors at the individual level in the family system may account for the variance of the development of child ODD symptoms. Additionally, individual parent and child factors, as proximal factors, are more inclined to play a moderated and mediated role in the association between distal family factors and child ODD symptoms in the family system. More studies that combine longitudinal and experimental design with moderator/mediator analyses are needed to further explore the interactive mechanism in the pathways from multilevel familial risk factors to child ODD symptoms.

### Limitations and Recommendations

We acknowledge several limitations in our review. First, we caution that the factors we have proposed to be related to child ODD symptoms are solely from family domains. We note that family domains are only part of the multiple pathways to the occurrence, maintenance, and development of child ODD symptoms. Although Bronfenbrenner (1979) considered the family system as the most proximal environment children confronted [110], there are possible risk factors that we need to consider in other domains (e.g., peer interaction, neighborhood environment, and social environment).

Second, we solely focused on the psychosocial factors. With strong biological correlates, additional factors, such as genetic, neurophysiological, neuroendocrine, and neuropsychological causes, should be underscored.

A third limitation, our study predominantly focused on the hierarchy of family factors at different levels and their effects on child ODD symptoms, the mutual linkages are not further elaborated in this review. However, the interplay of multilevel family factors and child ODD symptoms might initiate transactional feedback loops. Further research should underscore the reciprocal relationships between multi-level family factors and child ODD symptoms. Subsequently, although the selected literature addressed ODD or ODD symptoms, some of the literature also included CD/ODD or ODD with CD/ADHD. Therefore, we should be careful in our interpretation and comparison of data across studies. Future studies need to verify the three-level multiple family factors framework in the pure ODD group.

An increasing number of research studies have identified various moderators and mediators in the pathway to the development of child ODD symptoms. Over time, these efforts have identified prominently interactive effects among multilevel familial risk factors. Therefore, future researchers should pay more attention to the comprehensive and systematical mechanism underlying the occurrence of ODD symptoms. When considering the multiple pathways to ODD, in addition to carrying out research that replicates and expands upon the multiple risk factors, it is essential to examine the interactive effects among risk factors of multiple domains.

## 5. Conclusions

Overall, this review focused on the roles of familial risk factors in the etiology, maintenance, and development of ODD symptoms. These risk factors included lower family socioeconomic status (SES) and family dysfunction on the family system level; marital conflict and maladaptive parent–child relationship on the family dyadic level; individual parent factors, such as parental psychopathology, parental negative attribution style, parental emotion factors; and individual child factors, including difficult temperament, emotion dysregulation, impaired social cognition, and lack of empathy on the individual level. During the examination, the interactive mechanism among various risk factors was emphasized, which further accounted for the development of child ODD symptoms in the perspective of the family systems theory. Additionally, according to the review, when designing and developing family-based interventions and educational guidance to address child ODD symptoms, researchers and practitioners must lay an extreme emphasis on the dynamic and interactive effects of multiple levels of family factors that influence the emergence and exacerbation of ODD symptoms. As well, both the initial predictors and the mediators/moderators of child ODD symptoms should be taken into consideration, rather than focusing solely on the individual child-related factors.

## Figures and Tables

**Table 1:** Descriptive information of studies included in the review (*n* = 62).

Characteristic	*n*	% Study Sample
**Year of publication**		
2000–2010	20	32.3%
2011–2020	42	67.7%
**Methodology**		
Cross-sectional	19	30.7%
Longitudinal	43	69.3%
**Sample size**		
<100	8	12.9%
101–300	26	41.9%
301–600	12	19.4%
>600	16	25.8%
**Mean age of child participants**		
<5 years	16	25.8%
6–12 years	38	61.3%
13–18 years	8	12.9%

Note: The “Year of publication” indicates the year the article was published, the “Methodology” means whether the article was a cross-sectional study or a longitudinal study, the “Sample size” represents the sample size the study used, and the “Mean age of child participants” means the average age of the children in the article.

## Data Availability

Not applicable.
